# Supplementary material for: Impacts of the COVID-19 Pandemic on Cardiac Rehabilitation Delivery around the World
Source: Glob Heart. 2021 Jun 10;16(1):43. doi: 10.5334/gh.939 (PMC8195253; doi:10.5334/gh.939)
Supplement: Appendix 2. — Patient education mode and providers delivering, under usual practice and during COVID-19. [file gh-16-1-939-s2.pdf]

Appendix 2: Patient education mode and providers delivering, under usual practice and during COVID-19

Who delivers the education	**Under usual practice**	**During COVID-19**	**Not Offered**
Nurses	489 (73.1%)	101 (15.1%)	79 (11.8%)
Exercise specialist	486 (75.0%)	84 (13.0%)	78 (12.0%)
Healthcare providers from multiple disciplines	483 (75.8%)	56 (8.8%)	98 (15.4%)
Physician	250 (43.0%)	41 (7.0%)	291 (50.0%)
Mode of Delivery	**Yes, and continue to offer during COVID-19**	**Yes, but not during COVID-19**	**No**
Telephone-based sessions (individual or group)	385 (52.2%)	66 (8.9%)	287 (38.9%)
E-mailing resources as needed	360 (49.1%)	70 (9.5%)	303 (41.3%)
Online resources for patients to view/download (asynchronous)	352 (47.4%)	89 (12.0%)	302 (40.6%)
Mailing (postal service) resources as needed	341 (47.1%)	97 (13.4%)	287 (39.5%)
One-on-one sessions	280 (37.2%)	356 (47.3%)	117 (15.5%)
Virtual real-time/live interactive session (one-to-one)	143 (19.6%)	77 (10.6%)	508 (69.8%)
Virtual real-time/live sessions (group)	98 (13.4%)	90 (12.3%)	544 (74.3%)
Group sessions	79 (10.7%)	527 (71.5%)	131 (17.8%)
Other	28 (9.9%)	13 (4.6%)	243 (85.6%)

Note: frequency and valid percentage shown.
